# Micro-TESE in Non-Obstructive Azoospermia: Phenotype-Guided Hormonal Optimization and Testosterone Response—Prognostic Biomarker or Therapeutic Target?

**DOI:** 10.3390/jcm15155805

**Published:** 2026-07-24

**Authors:** Aris Kaltsas

**Affiliations:** Third Department of Urology, Attikon University Hospital, School of Medicine, National and Kapodistrian University of Athens, 12462 Athens, Greece; akaltsas@med.uoa.gr

**Keywords:** non-obstructive azoospermia, micro-TESE, sperm retrieval rate, intratesticular testosterone, clomiphene citrate, aromatase inhibitors, human chorionic gonadotropin, follicle-stimulating hormone, testosterone dynamics, male infertility

## Abstract

Non-obstructive azoospermia (NOA) is the most severe phenotype of male-factor infertility and reflects impaired spermatogenesis rather than ductal obstruction. Microdissection testicular sperm extraction (micro-TESE) is the primary sperm retrieval method, but sperm retrieval rates remain at approximately 40–60% and vary with etiology, genetics, histopathology, surgical expertise, and endocrine phenotype. This narrative review synthesizes major international and regional guidelines and contemporary evidence on preoperative hormonal optimization, with particular emphasis on endogenous testosterone dynamics. Exogenous testosterone is contraindicated in fertility-seeking men because it suppresses gonadotropins and intratesticular testosterone. By contrast, selective estrogen receptor modulators, aromatase inhibitors, human chorionic gonadotropin, and follicle-stimulating hormone aim to preserve or augment endogenous Leydig- and Sertoli-cell function. Low-certainty, predominantly observational evidence suggests an association between hormonal pretreatment and higher sperm retrieval in selected normogonadotropic or hypogonadal men, but not consistently in hypergonadotropic NOA. A larger testosterone rise during stimulation has been positively associated with retrieval and may reflect residual Leydig-cell reserve, although causality and transferable thresholds remain unproven. Preoperative endocrine therapy should therefore remain individualized, phenotype-guided, off-label, closely monitored, and preferably investigated within clinical trials; the testosterone response is best regarded as a candidate prognostic biomarker rather than a validated therapeutic target or decision rule.

## 1. Introduction

Non-obstructive azoospermia (NOA) is not a single pathological entity but the final common manifestation of severe quantitative or qualitative spermatogenic failure. The azoospermic ejaculate reflects the absence of spermatozoa in semen but does not prove the absolute absence of sperm production within the testis. In many men, spermatogenesis is spatially heterogeneous, with rare seminiferous tubules containing advanced germ cells surrounded by large regions of Sertoli-cell-only pattern, maturation arrest, hypospermatogenesis, or tubular sclerosis. This biological patchiness is the conceptual foundation of microdissection testicular sperm extraction (micro-TESE), which was developed to identify and selectively extract more promising tubules under high magnification while minimizing tissue loss [1,2].

The clinical stakes are high. A successful micro-TESE may enable intracytoplasmic sperm injection (ICSI) with autologous spermatozoa, whereas failure may direct the couple toward donor sperm, adoption, or discontinuation of treatment. Although micro-TESE is widely regarded as the preferred surgical retrieval method in NOA, success is not guaranteed: contemporary series report sperm retrieval rates (SRR) in the range of approximately 40–60% in experienced centers, with the individual probability varying by etiology, karyotype, Y-chromosome microdeletion status, testicular volume, endocrine pattern, prior gonadotoxic exposure, histology, and center expertise [2,3].

Within this uncertainty, preoperative hormonal optimization has become an attractive but controversial strategy. The rationale is straightforward: if residual spermatogenesis is present but suboptimally supported, pharmacologic manipulation of the hypothalamic–pituitary–gonadal (HPG) axis or direct gonadotropin stimulation might improve the intratesticular microenvironment before surgery. The controversy arises because NOA most often results from primary testicular failure, in which endocrine stimulation may be unable to overcome severe germ-cell depletion, and because the available evidence is dominated by retrospective cohorts, heterogeneous protocols, and selection bias rather than large randomized trials [4,5].

The central conceptual boundary is the difference between exogenous testosterone administration and pharmacologic enhancement of endogenous testosterone. Exogenous testosterone may normalize serum testosterone but suppresses luteinizing hormone (LH) and follicle-stimulating hormone (FSH), reduces intratesticular testosterone (ITT), and can induce or aggravate azoospermia. By contrast, clomiphene citrate, aromatase inhibitors, human chorionic gonadotropin (hCG), and FSH are used in selected fertility-seeking men to preserve or augment endogenous axis activity and intratesticular androgen exposure. Failure to distinguish these strategies leads to serious clinical misunderstanding, and it is reflected in the guideline position that testosterone therapy must not be used in men seeking fatherhood [6,7,8].

Accordingly, this narrative review examines the endocrine rationale, guideline boundaries, mechanism-based pharmacologic strategies, phenotype-specific clinical evidence, and the prognostic significance of endogenous testosterone dynamics in men with NOA undergoing micro-TESE. Throughout, a unifying argument is advanced: preoperative hormonal therapy in NOA is best framed not as empiric sperm induction but as phenotype-guided endocrine optimization coupled with functional testing of Leydig-cell reserve, in which the dynamic testosterone response is a candidate prognostic signal rather than a validated decision rule [9,10,11].

## 2. Evidence Acquisition: Search Strategy and Scope

This article is a narrative review and is not a systematic review conducted according to the Preferred Reporting Items for Systematic Reviews and Meta-Analyses (PRISMA) methodology. To enhance transparency and reproducibility, the search strategy and the criteria used to select the evidence are reported below, in keeping with recommended quality standards for narrative reviews (the Scale for the Assessment of Narrative Review Articles, SANRA).

The search emphasized publications from 2021 to 2026 to concentrate on evidence contemporaneous with the current guideline era and the most recent primary literature; foundational, mechanistic, and historical sources of enduring relevance—such as seminal descriptions of the testosterone-rebound phenomenon and core testicular physiology—were cited outside this window where appropriate.

**Data sources and search window.** PubMed/MEDLINE was searched, supplemented by Scopus and Embase where appropriate, together with the clinical practice guidelines of the European Association of Urology (EAU) and the American Urological Association/American Society for Reproductive Medicine (AUA/ASRM). The search emphasized articles published between January 2021 and June 2026, while seminal earlier studies relevant to micro-TESE technique, HPG-axis physiology, and preoperative testosterone optimization were retained irrespective of date.

**Search terms.** Search terms were combined with Boolean operators and included the following:

- “non-obstructive azoospermia” OR “NOA”;
- “micro-TESE” OR “microdissection testicular sperm extraction”;
- “sperm retrieval rate” OR “sperm retrieval”;
- “testosterone” OR “intratesticular testosterone” OR “testosterone response”;
- “clomiphene” OR “selective estrogen receptor modulator”;
- “aromatase inhibitor” OR “anastrozole” OR “letrozole”;
- “human chorionic gonadotropin” OR “hCG”;
- “follicle-stimulating hormone” OR “FSH” OR “gonadotropin”;
- “Klinefelter syndrome”; “hypogonadotropic hypogonadism”.

Targeted supplementary searches were also performed for regional clinical guidelines and reviewer-requested topics, including monogenic causes of NOA and differences in sex development, testicular imaging, prolactin and adrenal hormones, gonadotoxic cancer therapy, gender-affirming hormone therapy, and retinoic-acid-related approaches.

**Inclusion criteria.** Eligible sources included clinical practice guidelines; systematic reviews and meta-analyses; prospective and retrospective clinical studies reporting SRR or hormonal response; and studies pertinent to NOA, Klinefelter syndrome, hypogonadotropic hypogonadism (HH), or preoperative endocrine therapy before surgical sperm retrieval.

**Exclusion criteria.** Studies of isolated idiopathic oligozoospermia were excluded unless mechanistically relevant; non-human studies except where used solely to illustrate physiology; and sources without clinical relevance to fertility or sperm retrieval. Data presented only in conference-abstract form were not used as primary evidence for quantitative claims; where a peer-reviewed publication of the same work existed, the indexed article was cited instead.

**Evidence appraisal.** Each source was appraised for study design, population, intervention, endpoint definition, and risk of bias. Throughout the review, a distinction is drawn explicitly among established evidence, guideline recommendations, expert opinion, and biologically plausible but unproven hypotheses, and instances where causality cannot be inferred from association are noted.

**Use of generative artificial intelligence for figure preparation.** Google Gemini Apps (Nano Banana 2; Gemini 3.1 Flash Image; accessed on 21 July 2026) was used solely to generate and iteratively refine the schematic figures included in this article from author-defined scientific content and layout instructions. The author independently reviewed all labels, pathways, relationships, and captions against the cited literature and corrected any inaccuracies before inclusion. No generative AI tool was used to generate or analyze data, references, conclusions, or manuscript text.

## 3. Defining Non-Obstructive Azoospermia Before Hormonal Intervention

Rational use of preoperative hormonal therapy begins with correct classification of the patient. Misdiagnosis at this stage, whether overlooking rare ejaculated spermatozoa or mistaking obstruction for spermatogenic failure, changes the entire therapeutic pathway and may expose men to unnecessary pharmacologic or surgical intervention [6,12].

### 3.1. Confirming Azoospermia and Excluding Cryptozoospermia

Azoospermia should be confirmed on at least two semen analyses performed according to current World Health Organization methodology (WHO laboratory manual, 6th edition) [13], with examination of the centrifuged pellet. This distinction is not semantic: identification of rare spermatozoa in the pellet (cryptozoospermia) may permit cryopreservation of ejaculated gametes and can obviate or defer surgical retrieval. Failure to examine the pellet risks both overtreatment and an inaccurate denominator when counselling the couple about retrieval probability [6,12].

### 3.2. Differentiating Obstructive from Non-Obstructive Azoospermia

NOA is characterized by impaired sperm production, whereas obstructive azoospermia reflects preserved spermatogenesis with failure of transport. The distinction is supported by history (prior surgery, infection, cryptorchidism, chemotherapy, or exogenous androgen exposure), physical examination (testicular volume, vas deferens, epididymal distension), semen volume and pH, and the endocrine profile. Reduced testicular volume and elevated FSH favor NOA, but neither feature is specific enough to predict retrieval with certainty, and normal gonadotropins do not exclude severe spermatogenic arrest [6,12,14].

### 3.3. Genetic and Endocrine Work-Up

Before any intervention, evaluation should include karyotype and Y-chromosome microdeletion analysis, because complete AZFa or AZFb deletions carry a very poor retrieval prognosis whereas AZFc deletions are frequently compatible with sperm retrieval and ICSI [15,16], and because genetic findings carry implications for offspring counselling. Guideline statements on AZF deletions should nonetheless be applied with nuance: rare exceptions are documented, including a man with complete AZFb deletion but severe oligoasthenozoospermia who fathered a child by ICSI with ejaculated sperm and transmitted the deletion [17], and successful micro-TESE retrieval in partial AZFb deletion [18]; these involve partial deletions or residual/ejaculated spermatogenesis rather than sperm recovery in true complete-AZFb azoospermia, so the general prognostic rule stands while counselling accommodates individual variation. Beyond karyotype and Y-microdeletion testing, an expanding set of validated monogenic causes of NOA—including M1AP, STAG3, SYCP2, and TEX11—is associated with specific meiotic-arrest phenotypes [15,19], while congenital hypogonadotropic hypogonadism, including Kallmann syndrome, represents the hormonally distinct, gonadotropin-responsive end of the spectrum; a comprehensive account of NOA genetics is beyond scope [15]. The endocrine work-up comprises FSH, LH, total testosterone, calculated free testosterone or sex hormone-binding globulin, estradiol, and prolactin, with thyroid testing when indicated; inhibin B may be used as an adjunct. Prolactin is measured because hyperprolactinaemia causes secondary (hypogonadotropic) hypogonadism by suppressing GnRH-driven gonadotropin secretion, thereby impairing spermatogenesis, and represents a potentially treatable contributor; experimental data further indicate that both excess and deficient prolactin perturb spermatogenesis through effects on testicular steroid metabolism, although dedicated human evidence remains limited [20]. Testosterone should be sampled in the morning under standardized conditions and repeated when values are borderline or discordant with symptoms, and the calculated free value is particularly informative when total testosterone may be misleading. The aim is not merely to label the patient as hypogonadal but to identify a modifiable endocrine phenotype, recognizing the minority of men in whom a defined endocrine target exists for pharmacologic correction [6,11,14]. This diagnostic encounter also has value beyond fertility: NOA and impaired spermatogenesis are increasingly recognized as markers of broader health vulnerability, with population data linking severe male-factor infertility to higher all-cause mortality and to cardiometabolic and oncological risk, so the work-up doubles as an opportunity for long-term health risk stratification [21].

## 4. Histologic Heterogeneity and the Biological Rationale for Micro-TESE

NOA is not synonymous with the global absence of spermatogenesis. The testis in NOA typically displays a mosaic of histologic patterns, and it is this heterogeneity, rather than any uniform lesion, that micro-TESE is designed to exploit [1,2].

The principal histologic patterns are Sertoli-cell-only syndrome, in which tubules are lined exclusively by Sertoli cells; maturation (spermatogenic) arrest, in which germ-cell development halts at a defined stage and which may be sub-classified by the point of arrest as premeiotic (spermatogonial), meiotic (spermatocyte), or post-meiotic (spermatid); and hypospermatogenesis, in which all stages of spermatogenesis are present but markedly reduced. Peritubular fibrosis and tubular sclerosis frequently coexist, and mixed patterns are common within the same testis. Histology remains among the strongest available correlates of retrieval: hypospermatogenesis confers the most favorable probability, maturation arrest is intermediate and depends on whether arrest is early or late, and Sertoli-cell-only and sclerotic patterns are least favorable, although even these may harbor isolated sperm-producing tubules [1,2,22]. The same focal biology limits the predictive value of a separate diagnostic testicular biopsy: in a retrospective NOA series, diagnostic biopsy failed to detect spermatozoa in half of the men in whom sperm were subsequently recovered at therapeutic extraction, so a negative diagnostic biopsy should not deter micro-TESE and routine diagnostic biopsy before extraction is not recommended [23].

Microdissection exploits this focality directly. Under high magnification, seminiferous tubules that are larger and more opaque are more likely to contain active spermatogenesis and can be selectively excised, improving sperm yield while removing far less tissue than conventional biopsy [1,2]. This optical selection principle is also the mechanistic link to hormonal optimization, because treatments that enlarge active tubules may render them easier to identify intraoperatively, a hypothesis revisited mechanistically in Section 5 and Section 7.

This focal principle also explains the superiority of microdissection over conventional sampling. Conventional, non-microsurgical testicular sperm extraction (TESE) removes larger, randomly selected fragments and yields sperm in roughly 20–35% of men with NOA, whereas micro-TESE achieves approximately 40–60% by allowing the surgeon to identify, under magnification, the more promising seminiferous tubules, those appearing larger and more opaque, and to excise them within small tissue fragments while sparing the testicular microvasculature. By minimizing both the volume of tissue removed and vascular injury, microdissection is intended to protect Leydig-cell function and endogenous testosterone production, a consideration taken up in Section 11 [1,2].

The corollary is that no single preoperative variable reliably predicts retrieval. Systematic and meta-analytic evaluations of FSH, inhibin B, and anti-Müllerian hormone (AMH) have shown only modest discriminative ability, with AMH showing the most consistent signal but still insufficient accuracy to determine individual candidacy for surgery [22,24]. Paradoxically, in men with small testicular volumes, higher rather than lower FSH has been associated with successful retrieval, underscoring that conventional markers behave non-linearly across NOA subgroups [25]. Retrospective analyses likewise identify free and total testosterone, smoking status, and a history of prior TESE as factors associated with micro-TESE outcome, while again confirming that no combination of preoperative variables reliably determines success at the individual level [26]. These limitations motivate the search for functional, dynamic markers of testicular capacity that complement static baseline measurements [11].

## 5. The HPG Axis and the Critical Role of Intratesticular Testosterone

Spermatogenesis depends on a coordinated endocrine network. Pulsatile gonadotropin-releasing hormone (GnRH) from the hypothalamus drives pituitary secretion of LH and FSH. LH stimulates Leydig cells to synthesize testosterone, while FSH acts on Sertoli cells to support germ-cell survival, androgen-binding protein expression, metabolic provisioning, inhibin B production, and progression through the spermatogenic cycle. Testosterone and estradiol exert negative feedback at the hypothalamus and pituitary, and inhibin B selectively suppresses FSH [4,27].

### 5.1. Gonadotropin Signaling at the Cellular Level

**Figure 1:**
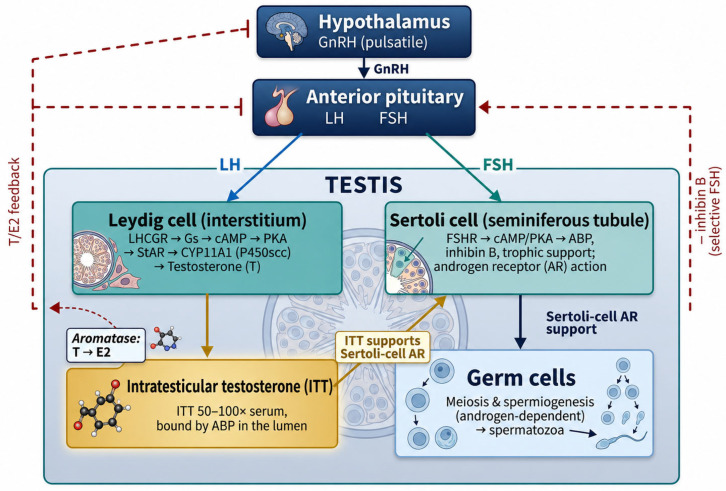
**The hypothalamic–pituitary–gonadal (HPG) axis and intratesticular testosterone (ITT).** Pulsatile GnRH drives pituitary secretion of LH and FSH. LH acts on the Leydig-cell LH/CG receptor (Gs-cAMP-PKA-StAR-CYP11A1) to synthesize testosterone, while FSH acts on Sertoli cells to produce androgen-binding protein, inhibin B, and trophic support. Locally produced testosterone is retained as intratesticular testosterone (50–100× serum) and, through Sertoli-cell androgen-receptor signaling, supports meiosis and spermiogenesis. Testosterone and its aromatized product estradiol exert negative feedback on the hypothalamus and pituitary, and inhibin B selectively suppresses FSH. Solid arrows denote stimulation; dashed blunted arrows denote negative feedback. Exogenous testosterone suppresses GnRH, LH, and FSH and lowers ITT (see main text).

At the cellular level, LH binds the LH/choriogonadotropin receptor, a G-protein-coupled receptor on the Leydig-cell membrane, activating adenylate cyclase and raising cyclic AMP, which through protein kinase A upregulates steroidogenic acute regulatory (StAR) protein. StAR mediates the rate-limiting transport of cholesterol into the mitochondrion, where the cholesterol side-chain-cleavage enzyme (CYP11A1) initiates the steroidogenic cascade that culminates in testosterone synthesis. FSH binds its own G-protein-coupled receptor on Sertoli cells, again signaling through cyclic AMP and protein kinase A to drive transcription of androgen-binding protein, inhibin B, and trophic factors that nourish developing germ cells and maintain the blood-testis barrier (Figure 1) [4,27].

Crucially, the androgen receptor (AR) that mediates the effect of testosterone on spermatogenesis is expressed in the nuclei of Sertoli and peritubular cells rather than in germ cells; testosterone therefore acts on the germ line indirectly, through Sertoli-cell AR signaling. Adequate intratesticular androgen acting on this receptor is required to complete meiosis and the differentiation of round spermatids during spermiogenesis, and a deficient androgenic signal arrests spermatogenesis at these steps [4]. Feedback is layered onto this system: testosterone and, importantly, its aromatized product estradiol suppress GnRH at the hypothalamus and LH and FSH at the pituitary, while Sertoli-cell-derived inhibin B selectively suppresses FSH. Because estradiol is a particularly potent central feedback signal, blocking estrogen action with a selective estrogen receptor modulator (SERM) or estrogen synthesis with an aromatase inhibitor disinhibits gonadotropin secretion; this is the molecular rationale shared by both drug classes [4,14,27].

### 5.2. Serum Testosterone Versus Intratesticular Testosterone

A defining feature of testicular physiology is that the seminiferous epithelium is exposed to androgen concentrations far higher than those measured in peripheral blood, on the order of 50- to 100-fold greater, sustained by androgen-binding protein within the tubular lumen. Serum testosterone is therefore a convenient but imperfect surrogate for the intratesticular androgenic milieu that drives meiosis and spermiogenesis. A man may exhibit adequate circulating testosterone yet insufficient intratesticular support, or, conversely, low serum testosterone while retaining focal intratesticular androgen production. This dissociation helps explain why a single serum testosterone value is an imperfect window onto the germinal compartment, a prognostic limitation developed in Section 10.1 [4,27]. Part of this dissociation is enzymatic: aromatase (CYP19A1), expressed mainly in Leydig cells, converts testosterone to estradiol, so the intratesticular androgen-to-estrogen balance—reflected clinically in the serum T/E2 ratio—depends on local aromatization; notably, the serum T/E2 ratio does not reliably reflect the intratesticular androgen-to-estrogen balance, cautioning against over-reliance on the serum value [28].

### 5.3. The Fertility Paradox of Exogenous Androgens

Exogenous testosterone disrupts fertility precisely because it replaces circulating androgen while removing the upstream drive for testicular androgen production. Testosterone therapy and anabolic-androgenic steroids reduce gonadotropin-releasing hormone pulsatility, suppress LH and FSH, lower ITT, and may produce severe oligozoospermia or azoospermia. For this reason, it is contraindicated in fertility-seeking men, the formal EAU and AUA/ASRM positions on which are detailed in Section 6 [7,8,9].

**Figure 2:**
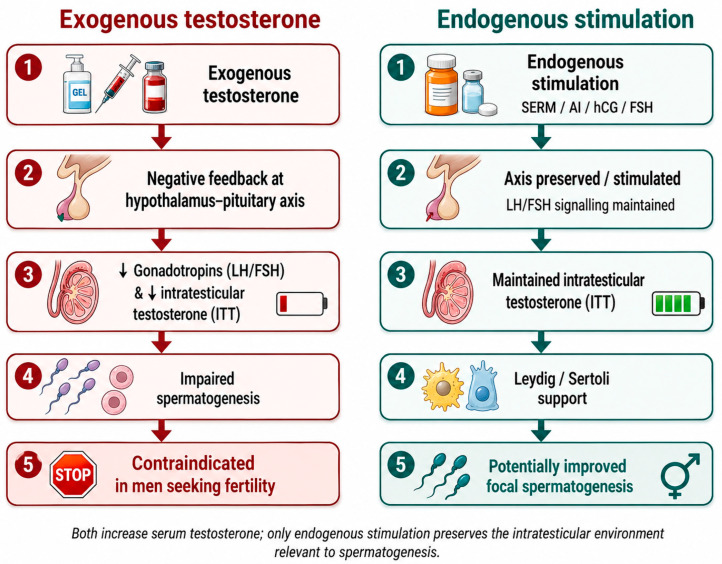
**Exogenous testosterone versus endogenous stimulation.** Schematic contrasting two pathways: (**left**) exogenous testosterone produces negative feedback, lowering GnRH, LH, and FSH, reducing intratesticular testosterone and impairing spermatogenesis; (**right**) selective estrogen receptor modulators, aromatase inhibitors, hCG, and FSH increase endogenous or gonadotropin-mediated Leydig- and Sertoli-cell support, potentially improving focal spermatogenesis.

The practical lesson is fundamental and frames the remainder of this review: raising serum testosterone is not equivalent to improving spermatogenesis. Fertility-oriented endocrine treatment seeks to preserve or stimulate ITT and Sertoli-cell function. Clomiphene citrate, aromatase inhibitors, and hCG can raise testosterone while maintaining or increasing LH and FSH signaling, whereas exogenous testosterone raises testosterone while suppressing the axis. This mechanistic divergence, not the serum testosterone value itself, separates fertility-preserving from fertility-suppressing strategies (Figure 2) [4,24,27].

This principle also has a historical dimension. In the mid-twentieth century, exogenous testosterone was deliberately administered to suppress and then, on withdrawal, to ‘rebound’ spermatogenesis in infertile men [29]. The approach was ultimately abandoned because the rebound was inconsistent and unpredictable—a cautionary precedent that reinforces the modern contraindication of exogenous testosterone in men seeking fatherhood.

### 5.4. Sertoli-Cell Function, the Blood-Testis Barrier, and Biomarkers of Testicular Function

The Sertoli cell is the structural and nutritive scaffold of spermatogenesis. Each Sertoli cell supports a finite number of germ cells, so the total Sertoli-cell population effectively sets the ceiling on sperm output. Adjacent Sertoli cells are joined near the basement membrane by a junctional apparatus that constitutes the blood-testis barrier, dividing the epithelium into a basal and an adluminal compartment; this barrier is not composed of tight junctions alone but of a composite of tight junctions—whose principal constituents include the claudins (notably claudin-11), occludin, and the scaffolding protein ZO-1—together with basal ectoplasmic specializations, desmosomes, and gap junctions [30]. The barrier creates an immunologically privileged adluminal space that sequesters meiotic and post-meiotic germ cells, which express stage-specific neo-antigens, from the immune system, and it sustains the specialized high-androgen microenvironment described above [4,27].

Sertoli cells also report on their own function through two secreted markers with clinical relevance. Inhibin B, produced in proportion to the integrity of Sertoli-germ cell interaction, both provides selective negative feedback on FSH and serves as a serum index of spermatogenic activity, whereas anti-Müllerian hormone reflects Sertoli-cell number and maturational state. These markers have been studied as non-invasive predictors of retrieval; meta-analytic data indicate that anti-Müllerian hormone carries the most consistent, though still individually insufficient, predictive signal, reinforcing that no single Sertoli-derived biomarker can determine candidacy for surgery [11,14,24].

### 5.5. Leydig-Cell Desensitization in the Hypergonadotropic State

The behavior of the Leydig-cell compartment explains why static testosterone measurements mislead in NOA. In primary testicular failure, chronically elevated LH drives downregulation and desensitization of the LH/CG receptor, through receptor internalization and reduced coupling efficiency, so that steroidogenic output per unit of LH falls. The result is the characteristic hypergonadotropic state, in which testosterone is low or low-normal despite markedly elevated LH, signifying compensated or overt Leydig insufficiency [4,27]. Because tonic endogenous drive is already maximal, the baseline serum testosterone reflects the prevailing balance rather than the residual capacity of the tissue. A dynamic challenge, captured as the testosterone increment during stimulation, instead probes the reserve that remains beyond this saturated endogenous drive, providing the mechanistic basis for interpreting the testosterone response discussed in Section 10. The same desensitization biology underlies the speculative proposal that transient suppression followed by re-stimulation might restore receptor sensitivity, a hypothesis that remains unproven [4,27]. More broadly, mechanistic reviews of Leydig-cell biology note that oxidative and endoplasmic-reticulum stress, mitochondrial dysregulation, apoptosis, and senescence-associated remodeling of the interstitial niche can erode steroidogenic capacity, framing the preservation of endogenous androgen production as a therapeutic objective [31].

## 6. Guideline Boundaries: Exogenous Testosterone Prohibition and Conditional Endogenous Stimulation

Because the field is frequently misrepresented as a simple choice between treating and not treating, it is essential to state precisely what the major guidelines prohibit, what they leave uncertain, and what they permit on a conditional basis (Table 1).

### 6.1. The EAU Position

The EAU maintains a strong recommendation not to use testosterone therapy to treat male infertility or in men who wish to father children. This is not a conservative preference without mechanistic basis; it follows directly from the biology of negative feedback and the dependence of spermatogenesis on ITT. In fertility-seeking men, exogenous testosterone should be regarded as a potential suppressor of spermatogenesis unless fertility is no longer relevant or gametes have already been banked [9].

The 2026 update of the EAU Sexual and Reproductive Health guideline makes this boundary explicit and extends it to the pre-surgical setting. Testosterone therapy is rated as contraindicated in infertile men (level of evidence 1a; strong recommendation), whereas spermatogenesis should be actively induced in men with congenital or acquired hypogonadotropic hypogonadism who wish to conceive, using hCG, human menopausal gonadotropin, or recombinant or highly purified FSH (strong recommendation) [9]. For the specific question addressed in this review, the guideline issues a distinct, weak recommendation that hormonal stimulation should not be started before testicular sperm extraction in men with NOA outside clinical trials, and concludes that no firm recommendation can be made for high-dose FSH in men with prior (micro-)TESE [9]. In classical (primary) testicular failure, the guideline finds no evidence that gonadotrophins, selective estrogen receptor modulators, or aromatase inhibitors improve spermatogenesis, consistent with the broader pharmacologic literature in which only testosterone replacement is advisable for primary hypogonadism and direct stimulation of Leydig-cell steroidogenesis is unreliable [9,32].

### 6.2. The AUA/ASRM Position

The AUA/ASRM guideline takes the same stance on exogenous androgens, stating that testosterone monotherapy should not be prescribed to men interested in current or future fertility. Importantly, however, the same guideline allows, as a conditional recommendation, that clinicians may use aromatase inhibitors, hCG, selective estrogen receptor modulators, or their combinations in infertile men with low serum testosterone. At the same time, it emphasizes that the evidence supporting pharmacologic manipulation specifically before surgical sperm retrieval in NOA is limited, and that patients should be counselled accordingly [7,8].

These two positions carry explicit but asymmetric evidence ratings: the option of endogenous stimulation in infertile men with low serum testosterone is a conditional recommendation (Grade C), whereas withholding exogenous testosterone from fertility-seeking men is stated as a clinical principle [7,8]. For NOA specifically, the guideline frames preoperative pharmacologic manipulation with selective estrogen receptor modulators, aromatase inhibitors, and gonadotropins as a discussion of limited supporting data rather than a directive to treat (conditional recommendation, Grade C), while micro-TESE is the recommended retrieval technique [7,8]. The practical importance of careful candidate selection is underscored by a multicentre cross-sectional analysis of 1644 men with NOA, in which only about 28% met endocrine criteria for clomiphene or hCG (total testosterone < 300 ng/dL with luteinizing hormone < 8 mIU/mL), 37% for aromatase inhibitors (T/E2 ratio < 10, with estradiol in conventional mass units), and roughly 18% for both, confirming that only a minority of men with NOA present a defined endocrine target for optimization [33].

These positions are broadly echoed—though with instructive regional and temporal variation—by more recent guidance. The 2023 Canadian Urological Association guideline advises stopping exogenous testosterone and, in its most directly relevant statement, issues a conditional (very-low-certainty) recommendation against neoadjuvant hormone therapy in testicular-failure NOA for the purpose of improving IVF-ICSI live-birth rates, while allowing that symptomatic hypogonadal men may still benefit from SERMs, aromatase inhibitors, or hCG [34]. The 2022 Urological Society of India guideline is comparatively more permissive of empirical medical therapy: it contraindicates testosterone replacement (strong recommendation) yet states that empirical hormonal treatment may be offered (moderate recommendation), specifically endorsing aromatase inhibitors for men with an abnormal T/E2 ratio, typically continued for two spermatogenic cycles before assisted reproduction [35]. The 2025 Japanese Urological Association guideline restricts hormonal agents by phenotype—gonadotropins for hypogonadotropic hypogonadism (grade A) and clomiphene citrate for oligozoospermia with low testosterone (grade B)—and positions micro-TESE (grade A) as the treatment for NOA without endorsing routine preoperative hormonal stimulation [36]. Thus, although these guidelines converge on withholding exogenous testosterone from fertility-seeking men, they diverge on empirical endogenous stimulation—from the explicitly cautionary Canadian position to the more permissive Indian one—reflecting shared evidentiary uncertainty rather than genuinely conflicting data.

### 6.3. The Interpretive Gap Between Guidelines and Practice

The apparent tension between conservative guidelines and widespread off-label practice is therefore narrower than it first appears. The guideline-consistent position is not that all hormonal therapy is forbidden; rather, exogenous testosterone is contraindicated, whereas stimulation of the endogenous axis remains selectively permissible but weakly evidenced. Guideline caution reflects the absence of adequately powered randomized trials, not a demonstration of harm from endogenous stimulation [4,5,10]; the field has aptly been characterized as one with too many rationales and too little data [37]. It must be stressed that this interpretation is not a recommendation to treat: the EAU explicitly advises against initiating hormonal stimulation before testicular sperm extraction in NOA outside clinical trials, and that position is endorsed here [9]. In expert andrology practice, particularly because micro-TESE is an end-of-line procedure with substantial psychological and financial weight, some clinicians nonetheless pursue a monitored trial of endogenous optimization in selected men; where this is done, it should occur preferably within a clinical trial or registry and otherwise only as explicitly documented, individualized off-label care, not as a de facto standard. The ethical requirement in either case is transparent informed consent: the patient must understand that such treatment is off-label, of uncertain benefit, and not a guarantee of sperm production [10].

Most recently, the Global Andrology Forum (GAF) provided NOA-specific clinical guidance through an international expert-consensus (Delphi) process, generating 49 graded recommendations and statements across diagnosis and treatment from a panel of 48 experts in 25 countries [38]. These statements are particularly relevant here because they address the same uncertainty highlighted by the EAU and AUA/ASRM: the absence of a universally applicable medical protocol before sperm retrieval and the need to correct reversible factors while avoiding indiscriminate hormonal therapy. In parallel, a worldwide GAF practice-pattern survey (336 respondents from 49 countries) documented substantial heterogeneity in preoperative hormonal use before surgical sperm retrieval, with such therapy recommended by only about 30% of respondents in normogonadotropic and 24% in hypergonadotropic men, confirming that current practice is driven by phenotype, local expertise, and clinician judgment rather than definitive randomized evidence [39].

The novelty of the present review lies not in restating these recommendations but in its focus. Comprehensive expert syntheses already map the evaluation and management of NOA in breadth—including the Global Andrology Forum recommendations and NOA guidance [38,39,40]—whereas this review is deliberately narrow: it examines the interpretation of the endogenous testosterone response as prognostic biomarker versus therapeutic target within a phenotype-driven optimization framework. Accordingly, adjacent topics such as the full genetic architecture of NOA, the spectrum of differences in sex development, oncological NOA, and gender-affirming therapy are acknowledged where relevant but treated concisely, with readers directed to dedicated sources.

## 7. Endogenous Hormonal Stimulation: Mechanistic Rationale and Therapeutic Classes

Endogenous stimulation aims to optimize the intratesticular androgenic environment and Sertoli-cell support while preserving, rather than suppressing, gonadotropin signaling; the underlying hypothalamic–pituitary–gonadal physiology and the central role of intratesticular testosterone are detailed in Section 5 and are not repeated here. Four pharmacologic classes, alone or in combination, are used toward this end; their selection should be driven by the endocrine phenotype rather than applied uniformly (Table 2) [4,27]. These agents form part of the wider empirical and off-label medical armamentarium for male infertility, the rationale and limitations of which have been reviewed elsewhere [41]; the basic and clinical rationale for hormonal therapy specifically in NOA has likewise been synthesized in dedicated reviews [42].

### 7.1. Selective Estrogen Receptor Modulators

Clomiphene citrate and tamoxifen act as estrogen-receptor antagonists at the hypothalamus and pituitary, attenuating estrogenic negative feedback and thereby increasing gonadotropin-releasing hormone pulsatility and endogenous LH and FSH secretion. The resulting LH drive raises testosterone produced locally within the testis, in contrast to exogenous androgen. The mechanistic rationale is strongest when gonadotropins are low or normal and weakest in severe hypergonadotropic failure, in which the axis is already maximally stimulated. Estradiol should be monitored, as exaggerated aromatization can offset the intended effect [4,14,27]. Clomiphene is a mixed estrogen-receptor agonist-antagonist whose dose is commonly titrated to a target serum testosterone; an early multicentre protocol titrated the dose toward a serum testosterone of approximately 600–800 ng/dL [50]. Recognized class effects, including visual disturbances and a rise in hematocrit with attendant thromboembolic concern, warrant monitoring of hematocrit and estradiol during therapy [4]. At the molecular level, clomiphene citrate is a racemic mixture of two isomers with divergent properties: enclomiphene, the predominantly antiestrogenic isomer responsible for gonadotropin stimulation, and zuclomiphene, a weak estrogen agonist with a long half-life that can accumulate with continued dosing; this composition shapes both the efficacy and the estrogenic adverse-effect profile of the drug [4]. Crucially, and in contrast to testosterone replacement, clomiphene raises testosterone without suppressing the hypothalamic–pituitary–gonadal axis, thereby preserving ITT and spermatogenesis, which is the property that makes it suitable for fertility-seeking men [51].

### 7.2. Aromatase Inhibitors

Anastrozole and letrozole inhibit the conversion of testosterone to estradiol, raising the testosterone-to-estradiol (T/E2) ratio and, secondarily, relieving estrogenic feedback to increase gonadotropins. The rationale is particularly relevant in men with a low T/E2 ratio or obesity-associated aromatization. Meta-analytic data confirm that aromatase inhibitors raise testosterone and the T/E2 ratio and lower estradiol, with improvements in conventional semen parameters in non-azoospermic men; excessive estradiol suppression is a potential limitation requiring monitoring [46]. The biological basis for this approach was established by the recognition of a treatable endocrinopathy in severe male infertility: affected men show a reduced T/E2 ratio (approximately 6.9 vs. 14.5 in fertile controls, reflecting lower testosterone and higher estradiol), which can be corrected by aromatase inhibition [52]. Aromatase is encoded by CYP19A1 and is expressed predominantly in adipose and hepatic tissue, accounting for the excess aromatization seen with obesity. A T/E2 ratio below approximately 10 is commonly used to select candidates for aromatase inhibition; this widely cited cut-off applies when testosterone is expressed in ng/dL and estradiol in pg/mL, and the numerical threshold necessarily differs when other units (for example, SI: nmol/L and pmol/L) are used, so the ratio should always be interpreted against the units and reference values of the reporting laboratory. Early protocols used testolactone, which has been largely superseded by the more potent and better-tolerated anastrozole and letrozole [52,53]. In a direct comparison of 140 subfertile men with an abnormal T/E2 ratio, anastrozole and testolactone produced similar improvements in the T/E2 ratio and semen parameters, with anastrozole achieving a better reduction in estradiol; the one exception was Klinefelter syndrome, in which testolactone appeared more effective, a nuance relevant to phenotype-specific selection [54].

### 7.3. Human Chorionic Gonadotropin

hCG shares an identical alpha-subunit with LH and acts on the same LH/CG receptor on Leydig cells, producing sustained stimulation of testosterone biosynthesis and an increase in intratesticular androgen, the determinant of spermatogenesis detailed in Section 5.2. The hCG beta-subunit, however, carries an additional carboxy-terminal peptide that markedly prolongs its circulating half-life, on the order of a day, compared with minutes for pituitary LH; hCG therefore provides sustained rather than pulsatile Leydig-cell stimulation. Its role is established in hypogonadotropic hypogonadism, where it can restore spermatogenesis, and it is used off-label in selected men with primary NOA to maximize the intratesticular androgenic milieu before retrieval [27]. Direct human evidence supports this mechanism: in men with NOA undergoing hCG-based therapy before a second micro-TESE, intratesticular testosterone rose roughly five-fold (from about 274 to 1348 ng/mL) and spermatogonial DNA synthesis increased significantly, indicating that the treatment acts on the germinal compartment and not merely on serum androgen [55]. Beyond steroidogenesis, hCG-based therapy has also been associated with reduced interstitial fibrosis and Leydig-cell hypertrophy, suggesting a remodeling of the testicular niche that may favor focal spermatogenesis [56].

### 7.4. Follicle-Stimulating Hormone and hMG

Recombinant FSH and human menopausal gonadotropin (hMG) act on Sertoli cells to support spermatogonial proliferation and maturation. The rationale is strongest in hypogonadotropic hypogonadism and is mechanistically contentious when endogenous FSH is already elevated, as in most hypergonadotropic NOA. Receptor-level considerations may nonetheless apply: FSH-receptor gene polymorphisms have been correlated with the seminal response to FSH therapy and have been proposed both to identify likely responders and to justify higher-dose regimens in selected men [57]. A related but speculative hypothesis holds that transient suppression followed by re-stimulation may “reset” desensitized gonadotropin receptors in the chronically hyperstimulated testis; this remains unproven and should be regarded as a mechanistic hypothesis rather than established practice [4,27].

Supportive evidence for the FSH class is strongest outside the azoospermic phenotype: in idiopathic infertile men with FSH in the normal range, meta-analysis indicates that FSH administration improves both spontaneous pregnancy rates and pregnancy rates after assisted reproductive technology, as well as sperm concentration, although the trials are heterogeneous, at high risk of bias, and lack validated criteria to guide patient selection [58]. Extrapolation of this signal to hypergonadotropic NOA is therefore not justified.

### 7.5. Combination Regimens

Combinations such as clomiphene plus hCG, hCG plus FSH/hMG, and an aromatase inhibitor plus hCG or a SERM are used to engage both Leydig-cell and Sertoli-cell signaling simultaneously. The theoretical advantage is more complete restoration of the intratesticular environment, but combination regimens also increase cost, monitoring burden, and the difficulty of attributing any observed effect to a single agent [4,27].

## 8. Clinical Evidence for Hormonal Therapy Before Micro-TESE

The mechanistic plausibility of endogenous stimulation must be weighed against the clinical evidence, which is heterogeneous and, for most agents, of low certainty, as emphasized in contemporary reviews of medical treatment before micro-TESE [59]. It is summarized below by therapeutic class (Table 3) before integrating the meta-analytic picture.

### 8.1. A Hierarchy of Clinical Endpoints

Before the evidence is appraised, the outcomes against which it is judged must be defined, because a recurrent weakness in this literature is the conflation of endocrine improvement with clinical success. A therapy that raises testosterone but does not increase usable sperm, embryo quality, or live birth should not be regarded as effective. A hierarchy of endpoints is therefore distinguished and applied throughout the sections that follow [5,10].

**Hormonal endpoint.** Increases in total and free testosterone, normalization of the T/E2 ratio, and changes in LH and FSH demonstrate biochemical activity but are surrogate measures only.

**Semen endpoint.** The appearance of spermatozoa in the ejaculate, including cryptozoospermia detected on the centrifuged pellet, is a meaningful intermediate outcome because it may permit cryopreservation and obviate surgery; gonadotropin therapy has achieved this in a minority of treated men [48,50,63].

**Surgical endpoint.** The sperm retrieval rate remains the principal outcome of micro-TESE, but it should be reported with attention to fresh versus frozen use, unilateral versus bilateral retrieval, and the amount of tissue removed. For this reason, the absolute retrieval rates cited throughout this review are not directly comparable across studies: they derive from heterogeneous populations and differ in denominator (per-patient versus per-procedure), in the definition of a positive result (any spermatozoon versus sperm usable for ICSI), and in fresh versus frozen use, so cross-study percentages should be read as context-dependent rather than as a ranking of efficacy.

**Reproductive endpoint.** Fertilization, embryo development, clinical pregnancy, and live birth are the outcomes that matter to couples. These are inconsistently reported, and at least one cohort that demonstrated a retrieval benefit reported no accompanying difference in clinical pregnancy or live birth, underscoring that improved SRR does not automatically translate into more children [48]. A further, frequently overlooked caveat is that live birth after testicular sperm and ICSI is governed at least as much by female factors, particularly maternal age and ovarian reserve, as by any male endocrine intervention; reproductive endpoints are therefore confounded across studies and cannot be attributed to preoperative hormonal therapy unless the female partner profile is accounted for.

### 8.2. SERMs Before Micro-TESE

Evidence for clomiphene is conflicting. An early multicentre protocol that titrated clomiphene (with hCG and hMG added for non-responders) toward target gonadotropin and testosterone levels reported sperm in the ejaculate in roughly 11% of men and a higher subsequent retrieval rate among those remaining azoospermic (57%) than in untreated controls (33.6%) [50]. More recent series have not confirmed a reproducible benefit: a retrospective study found micro-TESE outcomes unaffected by clomiphene [43], a cross-sectional study of clomiphene 25 mg daily for three months reported no increase (and a numerically lower retrieval rate) [44], and a 2026 non-randomized series of clomiphene plus hCG showed comparable retrieval (45% vs. 50%, not significant) despite a reduction in FSH [45]. Taken together, SERMs may have a role in selected hypogonadal or hormonally recruitable men but cannot be endorsed as a universal pre-micro-TESE protocol [4,5].

### 8.3. Aromatase Inhibitors Before Sperm Retrieval

The evidence for aromatase inhibitors is more convincing for endocrine and semen-parameter endpoints in non-azoospermic men than for retrieval in NOA. A meta-analysis of letrozole or anastrozole in men with a low T/E2 ratio demonstrated improved testosterone, T/E2 ratio, and sperm parameters [46], and multicentre cohort data indicate that responders tend to have favorable baseline hormonal profiles, whereas azoospermic men rarely respond [47]; the interpretation and clinical applicability of these predictors have been examined in accompanying commentary [64]. Direct evidence that aromatase inhibitors increase SRR at micro-TESE in unselected NOA remains limited [4].

### 8.4. hCG-Based Therapy

Gonadotropin-based regimens have shown signals of benefit in observational cohorts. In a single-center cohort of men with hypergonadotropic NOA, preoperative hCG with or without purified FSH was associated with a higher retrieval rate (31.2% vs. 19.5%; adjusted odds ratio [OR] 1.59) without differences in clinical pregnancy or live birth [48]. Proof-of-concept series of long-term recombinant gonadotropin therapy have reported the appearance of ejaculated or testicular sperm and occasional live births in men otherwise considered untreatable [65]. These data are encouraging but uncontrolled and subject to selection bias [27]. A seminal controlled observation comes from the salvage setting: among 48 men with a prior negative micro-TESE, 4–5 months of hCG (with recombinant FSH added if endogenous gonadotropins fell) before a second procedure yielded sperm in 6 of 28 treated men (21%) versus none of 20 untreated men, with success concentrated in those whose first histology showed hypospermatogenesis. This demonstrated that Leydig cells can respond to exogenous hCG even under hypergonadotropic conditions, providing a mechanistic proof of principle for gonadotropin stimulation in primary NOA [66].

### 8.5. FSH and Gonadotropins

A clear distinction must be drawn between hypogonadotropic hypogonadism, in which gonadotropin therapy is etiologic and frequently restores spermatogenesis, and hypergonadotropic NOA, in which it is empirical and contested. A prospective assessment of recombinant gonadotropin therapy in NOA found benefit concentrated in men with late maturation arrest, reinforcing that any effect is phenotype-dependent rather than general [49]. Evidence in the azoospermic setting specifically is sparse and low-grade: a small non-randomized controlled study of highly purified FSH (urofollitropin) before extraction in idiopathic azoospermia reported modest increases in pregnancy and fertilization rates but no convincing gain in retrieval, while gonadotropin combinations have improved the retrieval rate in only isolated NOA cohorts [50,67]. An older controlled study of pure FSH before extraction in normogonadotropic NOA reported a higher overall retrieval rate in treated than untreated men (64% vs. 33%), with the benefit concentrated in hypospermatogenesis and focal spermatogenesis and absent in Sertoli-cell-only histology, again indicating that any effect is histology- and phenotype-dependent rather than universal [68]. The guideline synthesis accordingly confines gonadotropin use before extraction in NOA to clinical trials rather than routine care [9].

### 8.6. Meta-Analytic Synthesis

The most informative synthesis is the systematic review and meta-analysis of hormonal therapy before surgical sperm retrieval, which pooled 22 studies and 1706 participants. Overall, pre-treatment was associated with higher retrieval (OR 1.96, 95% confidence interval [CI] 1.08–3.56). Crucially, the subgroup analysis showed a significant benefit only in normogonadotropic men (OR 2.13, 95% CI 1.10–4.14, *p* = 0.02) and not in hypergonadotropic men (OR 1.73, 95% CI 0.44–6.77, *p* = 0.43), with the literature at moderate-to-severe risk of bias and low overall certainty [5]. A complementary pooled analysis found that men with normal baseline testosterone had higher retrieval than those with subnormal testosterone (OR 1.63, 95% CI 1.08–2.45), suggesting that the patients most likely to benefit are those with preserved or recruitable endocrine function rather than profound primary failure [4]. The consistent conclusion across syntheses is that hormonal therapy should not be used routinely in unselected NOA before micro-TESE, and that adequately powered randomized trials are needed [4,5,10]. Earlier focused appraisals of endocrine stimulation before surgical retrieval reached the same verdict, emphasizing the paucity of controlled safety and efficacy data despite widespread use [69].

### 8.7. Combination Protocols

Data on combination regimens are limited but suggestive. In idiopathic infertile men, combined clomiphene citrate and anastrozole duotherapy achieved normozoospermia more often than anastrozole monotherapy (43% versus 25%), with gains in total motile sperm count, indicating additive effects on the endocrine milieu [70]. In hypergonadotropic NOA due to maturation arrest, a sequential strategy of pituitary desensitization with a GnRH agonist followed by menotropin stimulation produced ejaculated spermatozoa and two live births, offering a potential non-surgical route in a highly selected setting [71]. These reports are small and uncontrolled; combination therapy adds cost and monitoring burden and should be reserved for clearly reasoned, individualized indications.

## 9. Phenotype-Specific Interpretation: Which NOA Patients May Be Endocrinologically Recruitable?

If hormonal therapy benefits some men but not unselected NOA as a whole, the central clinical question becomes which phenotypes are plausibly recruitable. Organizing the evidence by phenotype, rather than by drug, converts a pharmacologic list into a usable decision framework [4,5,10]. A complementary effort to standardize this stratification is the APHRODITE classification, an expert-consensus system that partitions infertile men into hypogonadotropic, normogonadotropic-eugonadal, normogonadotropic-hypogonadal, hypergonadotropic, and unexplained subgroups, explicitly to align candidate selection and future hormonal-therapy trials [72].

### 9.1. Hypogonadotropic Hypogonadism

Hypogonadotropic hypogonadism is the one setting in which therapy is etiologic rather than empirical. Congenital hypogonadotropic hypogonadism is itself genetically heterogeneous and frequently oligogenic, with variable penetrance and expressivity across the pathways governing GnRH-neuron development, migration, secretion, and action; this genetic architecture underlies the phenotypic spectrum and the variable, though generally favourable, response to gonadotropin therapy [73]. Stepwise hCG, with or without FSH, frequently induces spermatogenesis and may restore sperm to the ejaculate, reserving micro-TESE for persistent azoospermia. In a contemporary cohort of men with HH-related NOA, gonadotropin therapy produced ejaculated sperm in 77% of men, micro-TESE retrieved sperm in 88% of those remaining azoospermic, and approximately half ultimately achieved biological fatherhood [63]. This phenotype should always be identified before surgery, as it may render micro-TESE unnecessary. Meta-analytic data in hypogonadotropic hypogonadism indicate that gonadotropin therapy achieves spermatogenesis in approximately three-quarters of treated men, with better outcomes in post-pubertal-onset disease, lower baseline gonadotropins, combined hCG plus FSH, and greater testicular volume, whereas the FSH preparation used does not materially affect the result [74]. Larger testis volume, prior gonadotropin exposure, and the avoidance of prior androgen therapy independently predict faster induction of spermatogenesis and pregnancy [75]; notably, previous testosterone replacement does not appear to compromise the eventual response [74]. Response is not, however, universal: NR0B1 (DAX1) mutations are a recognized exception in which spermatogenesis is impaired and gonadotropin therapy is frequently unsuccessful, although TESE-ICSI may still offer paternity [76].

### 9.2. Hypogonadal NOA with Low Serum Testosterone

Men with primary NOA and low serum testosterone are the principal target for endogenous stimulation. Here the relevant question is not the baseline value but whether residual Leydig-cell capacity can be recruited, a concept developed in the next section. Cohort data in hypogonadal NOA suggest that hormonal stimulation and the resulting testosterone response are associated with retrieval, although causality is not established [10]. A reported case of a hypogonadal man with normal serum 17-hydroxyprogesterone, used as a surrogate for ITT, illustrates that preserved intratesticular androgen production can coexist with low serum testosterone and support successful retrieval despite an apparently unfavorable serum profile [77].

### 9.3. Low T/E2 Ratio and Obesity-Associated Aromatization

A low T/E2 ratio, often associated with obesity, identifies men in whom aromatase inhibition is mechanistically appropriate. Treatment raises testosterone and the T/E2 ratio and lowers estradiol, with the caveat that responders tend to have more favorable baseline gonadotropin profiles and that azoospermic men respond less reliably than oligozoospermic men [46,47]. In a small randomized, placebo-controlled pilot study confined to azoospermic and cryptozoospermic men with a T/E2 ratio below 10, letrozole significantly increased sperm concentration, with the response inversely related to baseline T/E2, FSH, and body-mass index and directly related to testicular volume, illustrating both the phenotype-dependence and the modest magnitude of any effect [78]. Estradiol must be monitored to avoid over-suppression.

### 9.4. Hypergonadotropic NOA

Elevated FSH and LH define the most common NOA phenotype and the one least likely to be pharmacologically reversible, because the axis is already maximally stimulated. Importantly, high gonadotropins do not exclude retrieval, and selected gonadotropin-based regimens have yielded isolated positive signals in this group; however, the meta-analytic data summarized in Section 8.6 do not support a routine effect, and therapy should be considered only selectively and within a monitored framework [5,48].

### 9.5. Klinefelter Syndrome

The pathophysiology of non-mosaic Klinefelter syndrome (KS) explains both its distinctive endocrine profile and its time-dependent prognosis. The supernumerary X chromosome is associated with accelerated germ-cell loss and progressive hyalinization and fibrosis of the seminiferous tubules that intensifies from the onset of puberty, while the interstitium shows Leydig-cell hyperplasia with paradoxically impaired function, yielding a hypergonadotropic state with relative androgen deficiency and a characteristically low T/E2 ratio. The net consequence is a testis that retains only scattered foci of preserved spermatogenesis within a largely sclerotic parenchyma, which both accounts for the decline in retrieval with advancing age and provides the rationale for early intervention and for androgen-environment optimization rather than exogenous testosterone [3,79,80]. The endocrine phenotype may extend beyond the gonadal axis: detailed characterisation of Klinefelter syndrome and higher-grade aneuploidies has shown reduced DHEA-S alongside the expected decline in testosterone and inhibin B [81], though a direct mechanistic link between adrenal androgens and spermatogenesis remains hypothesis-generating.

Non-mosaic Klinefelter syndrome therefore warrants separate consideration: small testes, hypergonadotropic hypogonadism, and progressive tubular degeneration coexist with focal residual spermatogenesis in a subset of men, and exogenous testosterone must be avoided until the fertility pathway is complete. Retrieval rates vary widely with cohort and technique. In a large multicentre real-life series of non-mosaic KS, the retrieval rate was 29.8%, with higher baseline total testosterone and hypospermatogenesis on histology emerging as independent predictors [61]. A retrospective comparison reported an overall retrieval rate of 32.6% after preoperative therapy, with anastrozole associated with more favorable outcomes than choriogonadotropin alfa despite comparable testosterone increases [53]. By contrast, a single-center cohort treated with recombinant hCG before micro-TESE reported a markedly higher retrieval rate of 73.7%, within which the testosterone response, testicular volume, and age structured a decision tree for prognosis [62]. This 73.7% is a clear outlier, roughly double the rates in the larger multicentre and comparative series above, and likely reflects center- and protocol-specific factors and denominator differences rather than a generalizable effect; it should be weighted accordingly. The heterogeneity across these series underscores that absolute retrieval rates in KS are center- and protocol-dependent, while the prognostic role of the testosterone response is comparatively consistent.

Other differences in sex development—including 45,X/46,XY mixed gonadal dysgenesis, 46,XX testicular DSD, and testicular dysgenesis—may also present with NOA and hypergonadotropic hypogonadism. In an unselected cohort of 865 azoospermic men, loss of Y-chromosome material spanning the spectrum from AZF microdeletions to complete loss of the Y chromosome was found in 6.1%, including 45,X and 45,X/46,XY mosaic karyotypes, and partial AZFb microdeletions were associated with a milder testicular phenotype than complete AZFb deletions [82]. A full account of these conditions is beyond this review’s scope.

### 9.6. Prior Failed Micro-TESE (Salvage Setting)

Men with a previously failed micro-TESE constitute a distinct, high-stakes subgroup in which repeat surgery carries lower expected yield and greater risk to testicular endocrine function. Retrieval rates fall substantially with repetition: in a retrospective cohort, first-time micro-TESE achieved sperm retrieval in 64.6% of men compared with 28.8% for repeated procedures (*p* < 0.01), and repeat cases were older with higher FSH and LH and lower testosterone, an adverse profile suggesting limited recruitable reserve [83]. Evidence specific to preoperative hormonal therapy in this setting is sparse, and any attempt should be reserved for men whose prior histology demonstrated hypospermatogenesis or late maturation arrest, after explicit counselling about the reduced probability of success [2,5,83]. A history of exogenous androgen exposure—including testosterone given in childhood or adolescence for hypogonadism—may further suppress an already compromised axis; however, prior testosterone-induced virilization does not necessarily preclude subsequent testicular growth and spermatogenesis, as a multicentre prospective study showed that hCG combined with recombinant FSH induced testicular growth and spermatogenesis in adolescents with hypogonadotropic hypogonadism, albeit often requiring 12–24 months or longer [84]. Even when prolonged gonadotropin therapy fails to produce ejaculated sperm, subsequent micro-TESE may still succeed [85]. Evidence on the optimal timing of testosterone therapy relative to fertility preservation nonetheless remains mixed, particularly in Klinefelter syndrome, where recommendations on sperm preservation in adolescence continue to conflict [86,87,88]. Any endocrine “salvage” attempt in this setting therefore remains individualized and of unproven benefit and should follow explicit counselling about the reduced probability of success.

## 10. Endogenous Testosterone Dynamics: Biomarker, Mediator, or Epiphenomenon?

The thread connecting the preceding sections is the possibility that the response of endogenous testosterone to stimulation, rather than any static value, carries prognostic meaning. This is the conceptual core of the present review, and it must be argued carefully to avoid overstatement [10,11]. This caution is consistent with the wider evidence on retrieval prediction, in which no single clinical, hormonal, or histologic variable reliably forecasts success and multivariable integration remains the most realistic path forward [11].

### 10.1. Baseline Testosterone Versus the Stimulated Response

Baseline serum testosterone has shown inconsistent prognostic performance across NOA cohorts. In a large historical cohort, men with baseline testosterone above and below 300 ng/dL had statistically indistinguishable retrieval rates (56% vs. 52%, *p* = 0.29), and neither the baseline value nor the presence of a response to therapy altered sperm retrieval, clinical pregnancy, or live birth [89]. In specific phenotypes, however, baseline testosterone retains predictive value, as in non-mosaic Klinefelter syndrome (Section 9.5); this heterogeneity, rather than a uniform absence of signal, motivates the shift toward a dynamic measure of Leydig-cell capacity.

### 10.2. Delta-Testosterone as a Functional Test of Leydig-Cell Reserve

The magnitude of the testosterone increase during stimulation (delta-testosterone) can be conceptualized as an indirect clinical stress test of Leydig-cell reserve, the depletion of which in the chronically hyperstimulated testis was introduced in Section 5.5. In a cohort of hypogonadal men with NOA, both the post-treatment testosterone and the absolute rise from baseline were associated with retrieval (Table 4), with a pre-micro-TESE testosterone of approximately 418.5 ng/dL (area under the curve [AUC] 0.78) and a delta-testosterone of approximately 258 ng/dL (AUC 0.76) distinguishing positive from negative outcomes; the authors explicitly stated that causality was not established [10]. In an independent series of men with NOA and low testosterone treated with an aromatase inhibitor or recombinant hCG, both the pre-operative testosterone and the testosterone change differed significantly between retrieval-positive and retrieval-negative men, with a receiver-operating characteristic area under the curve of 0.785 [60]. Reported thresholds are not directly comparable across studies because of differing assays and units (ng/dL versus nmol/L), a limitation revisited below.

### 10.3. Relationship with the Intratesticular Microenvironment

**Figure 3:**
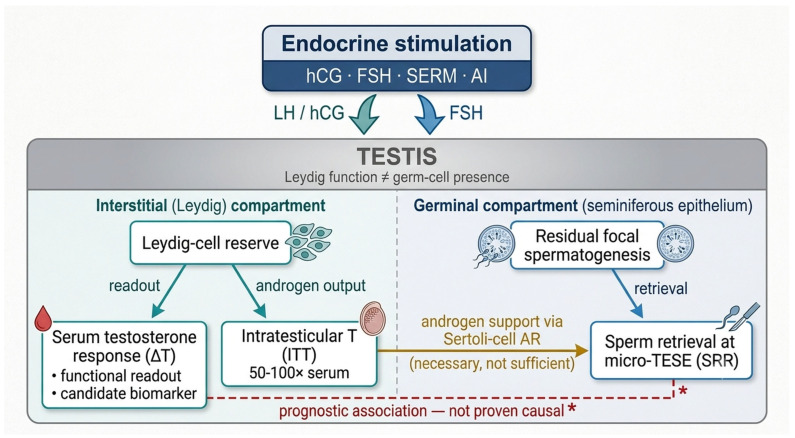
**Testosterone dynamics as a functional biomarker (conceptual model).** Endocrine stimulation acts on the interstitial (Leydig) compartment, whose reserve is read out as the rise in serum testosterone (delta-testosterone) and sustains intratesticular testosterone. The germinal compartment, in which residual focal spermatogenesis determines sperm retrieval at micro-TESE, is shown separately to emphasize that Leydig-cell function and germ-cell presence are related but not equivalent. Solid arrows denote physiological support; the dashed arrow denotes a prognostic association between the testosterone response and sperm retrieval that is not proven to be causal.

A robust testosterone response plausibly indicates that functional Leydig cells remain and can generate a favorable intratesticular androgenic milieu, whereas a blunted response may reflect advanced interstitial fibrosis and depleted Leydig-cell capacity. Critically, Leydig-cell function and germ-cell presence are related but not equivalent: a man may mount a strong testosterone response yet still lack retrievable germ cells, because the response reports on the interstitium rather than on the seminiferous epithelium (Figure 3) [10,11]. The difficulty of measuring the intratesticular compartment directly has prompted interest in serum surrogates: serum 17-hydroxyprogesterone, a Leydig-cell steroid intermediate, has been proposed as an indirect marker of ITT and may help identify men with preserved intratesticular androgen production despite low serum testosterone [77]. At the tissue level, Sertoli-cell androgen-receptor expression is upregulated by FSH-based therapy and rises more in men in whom sperm are subsequently retrieved, linking the androgen-signaling response to retrieval; intriguingly, men with successful retrieval have shown lower basal intratesticular testosterone yet greater treatment-induced germ-cell proliferation, implying that the capacity to respond may matter more than the absolute baseline value [55,90]. Consistent with this mechanism, Leydig-cell aromatase expression is increased in NOA—particularly in maturation-arrest and Sertoli-cell-only phenotypes—and higher aromatase, accompanied by lower intratesticular testosterone and a lower intratesticular T/E2 ratio, is independently associated with a lower probability of sperm retrieval at micro-TESE; serum and intratesticular T/E2 do not correlate, and aromatase inhibition (anastrozole) raises intratesticular testosterone and the T/E2 ratio, providing a rationale for a therapeutic window in selected men [28].

### 10.4. Correlation Versus Causation

This distinction is the crux of the interpretive problem. An association between the testosterone response and retrieval does not establish that hormonal therapy caused sperm production; the response may simply mark a less severely damaged testis that was always more likely to yield sperm. The available data are observational and cannot separate a predictive (prognostic) interpretation from a causal (therapeutic) one. The testosterone response may therefore identify a biologically favorable phenotype without proving that augmenting testosterone changes the outcome [5,10].

### 10.5. The Klinefelter Paradigm

Klinefelter syndrome provides the clearest illustration of the response as a prognostic axis. In a decision-tree analysis of non-mosaic KS treated with recombinant hCG, the testosterone change was the primary determinant of retrieval, testicular volume a secondary contributor, and age a tertiary consideration; the highest success (approximately 97%) occurred in men with a large testosterone rise and preserved testicular volume, and the lowest (approximately 63%) in older men with no testosterone rise and small testes [62]. These figures must be read with caution: they come from the same single-center series whose overall retrieval rate (73.7%) is roughly double that of larger cohorts, so the absolute success percentages are almost certainly center-specific and not transferable, even though the direction of the signal, a larger testosterone response tracking with higher retrieval, is internally consistent and echoed by independent real-life data that confirm baseline total testosterone as an independent predictor in KS [61]. Taken together, these findings position the testosterone response as a candidate stratification tool whose ranking value may generalize but whose absolute thresholds and rates still require external validation.

### 10.6. Integration into Multivariable Prediction

No single marker, including the testosterone response, should be used in isolation. The response is best integrated with age, testicular volume, baseline FSH and LH, baseline testosterone, estradiol and the T/E2 ratio, inhibin B and AMH, histology where available, karyotype and Y-chromosome microdeletion status, etiology, and any history of prior retrieval. Contemporary efforts to build and validate multivariable models and nomograms, increasingly incorporating dynamic endocrine variables, represent the most promising path toward individualized prediction [11,22,24]. Static indices of Leydig-cell efficiency may also complement dynamic endocrine testing: the testosterone-to-LH ratio reflects Leydig-cell output relative to pituitary drive and, in a retrospective cohort of men with NOA undergoing micro-TESE, was independently associated with successful retrieval together with FSH and testicular volume [91]. Such static indices should not replace the stimulated testosterone response, but they help characterize whether a given baseline testosterone is being maintained with low, appropriate, or excessive LH drive. Emerging molecular markers may eventually complement such models; for example, testicular small-RNA (microRNA) signatures have been associated with sperm presence and post-retrieval outcomes in azoospermic men, although these markers remain at the discovery stage [92]. Serum hormones should not be interpreted in isolation but alongside testicular volume and imaging: scrotal ultrasonography—including elastographic measures of testicular stiffness—combined with volume has shown moderate predictive value for retrieval before micro-TESE (area under the curve ~0.74), with magnetic-resonance imaging reserved for selected cases [93,94]. Beyond volume alone, ultrasonographic assessment of the seminiferous tubules themselves has been incorporated into prediction: a model integrating the ultrasonographically determined seminiferous tubule diameter with age, serum FSH, a history of cryptorchidism, and sex-chromosome abnormalities predicted sperm retrieval at micro-TESE with useful discrimination [95], and high-frequency ultrasonography of the seminiferous tubules has since shown correlation with testicular histology [96]. In parallel, quantitative diffusion-weighted MRI—specifically volumetric apparent-diffusion-coefficient histogram analysis—has been proposed as a non-invasive imaging fingerprint of impaired spermatogenesis [97]. These emerging non-invasive phenotyping tools remain investigational and require prospective external validation before they can refine candidate selection. Nonetheless, no single parameter, endocrine or radiological, reliably excludes retrieval, and these tools refine counselling rather than determine candidacy.

## 11. Clinical Application: Algorithm, Surgical Endocrine Cost, and Off-Label Prescribing

This section translates the preceding evidence into practice. It first sets out a phenotype-driven algorithm for selecting and managing candidates, then addresses the practical dimensions that surround the procedure itself: the endocrine cost of the retrieval technique, the consequences of surgical failure and repeated attempts, and the safety and ethical obligations of off-label prescribing.

### 11.1. A Phenotype-Driven Algorithm

**Figure 4:**
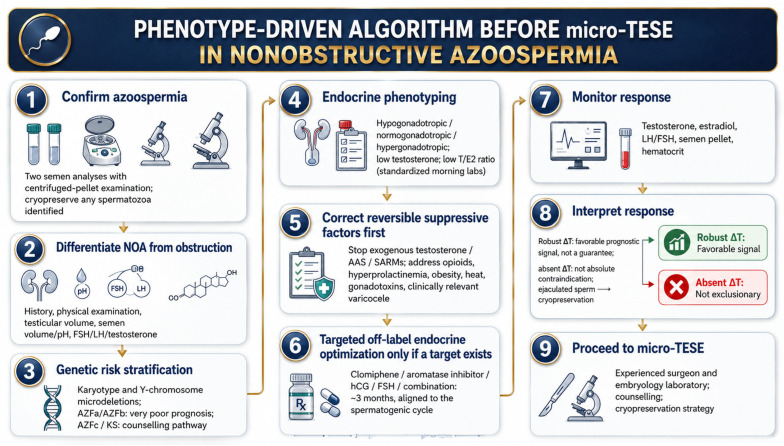
**Phenotype-driven algorithm before micro-TESE.** Flow from confirmation of azoospermia through genetic and endocrine phenotyping to a decision on whether a pharmacologic target exists, followed by correction of reversible suppressive factors, monitored optimization, reassessment of testosterone, estradiol, and the semen pellet, cryopreservation of any sperm, and progression to micro-TESE.

Synthesizing the preceding evidence, a stepwise, phenotype-driven approach is proposed to identify the minority of men with a defined endocrine target while avoiding indiscriminate treatment; the complete pathway, from confirmation of azoospermia through phenotyping, correction of reversible factors, monitored optimization, and reassessment to micro-TESE, is shown in full in Figure 4 [4,6,11].

In outline, once azoospermia and its non-obstructive nature are confirmed and genetic and endocrine phenotyping are complete, reversible suppressive factors, above all exogenous androgen exposure, are corrected first. Pharmacologic stimulation is then offered only when a modifiable endocrine target is present and for a defined, monitored interval of approximately three months, with the testosterone response read as a functional signal rather than a decision rule: favorable when robust, yet not an absolute contraindication when absent. Any spermatozoa appearing in the ejaculate during treatment are cryopreserved immediately; otherwise the patient proceeds to micro-TESE with an experienced surgical and embryology team and a defined cryopreservation strategy.

Reversible non-hormonal contributors should be addressed in the same step before a patient is labelled endocrinologically non-recruitable. In men with a palpable clinical varicocele, a systematic review and meta-analysis of azoospermic men found that repair improved sperm concentration, progressive motility, and morphology, although serum FSH and LH did not change significantly and the evidence was heterogeneous [98]. Notably, only a minority of azoospermic men regain sperm in the ejaculate after varicocele repair, and the decision to intervene must weigh the low probability of spontaneous conception against surgical effort and the timing of micro-TESE [99]. Varicocele correction should therefore be viewed as part of broader preoperative optimization rather than as a substitute for endocrine phenotyping or micro-TESE planning.

### 11.2. Micro-TESE Versus Conventional TESE and Testicular Preservation

The value of optimizing the testis before surgery is amplified by the recognition that retrieval itself is not endocrinologically neutral. Beyond the retrieval-yield advantage of microdissection over conventional TESE discussed in Section 4, the two techniques differ in their endocrine cost: conventional, non-microsurgical TESE removes larger, randomly chosen fragments and disrupts testicular blood supply and Leydig-cell mass, whereas microdissection permits selective excision of small tissue fragments under magnification with preservation of the intratesticular vasculature, limiting the amount of tissue removed and the consequent endocrine injury [1,2].

### 11.3. Post-Operative Hypogonadism and the Repeated-Retrieval Setting

Even micro-TESE carries a measurable endocrine cost. In a nested case-cohort of men followed after failed bilateral micro-TESE, total testosterone fell by a mean of 73 ng/dL (16%) and remained durably reduced, and 36% of men who were eugonadal preoperatively became biochemically hypogonadal, accompanied by a compensatory rise in FSH [100]. This single-cohort signal is corroborated by meta-analytic evidence: a systematic review and meta-analysis of 17 studies including 1685 azoospermic men found a significant fall in total testosterone after surgical sperm retrieval (mean difference 3.81 nmol/L), whereas postoperative changes in FSH, LH, and testicular volume were not statistically significant [101]. The cost compounds with repetition: retrieval falls from 64.6% at first micro-TESE to 28.8% at repeat surgery, and repeat-procedure men display older age and an adverse hormonal profile consistent with diminished reserve [83]. Micro-TESE should therefore be regarded not only as a retrieval procedure but also as a potential endocrine stressor, particularly in men with limited Leydig-cell reserve or those considered for repeat retrieval. These observations strengthen the rationale for careful candidate selection, for techniques that preserve testicular function, and for considering the endocrine context, rather than the retrieval probability alone, when planning surgery.

### 11.4. Off-Label Prescribing, Counselling, and Monitoring

Because endogenous stimulation in NOA is off-label, its use carries specific safety and ethical obligations. Patients must be told that the strategy is unproven for increasing sperm retrieval, that exogenous testosterone is contraindicated, and that treatment must not delay definitive micro-TESE beyond a defined window. Clomiphene and aromatase inhibitors require monitoring of estradiol and hematocrit and carry recognized adverse effects, while hCG and FSH add injection burden and cost. The indication and the measured response should be documented. The appropriate framing is explicit: off-label hormonal optimization is a monitored attempt to improve endocrine conditions before surgery, not a proven method to generate sperm in men with irreversible spermatogenic failure [7,8,9,10]. Precision of language is part of this obligation: it is inaccurate to state that hormonal therapy “restores fertility” in NOA, whereas it is defensible to say that it may optimize endocrine conditions before micro-TESE in selected patients while the evidence remains mixed. This phrasing preserves realistic hope without overstating efficacy and aligns with the duty not to delay definitive care on unproven assumptions [10].

### 11.5. Synthesis and Overall Position

Drawing the preceding evidence together, three conclusions can be stated with reasonable confidence. First, exogenous testosterone is contraindicated in fertility-seeking men, on mechanistic grounds not in dispute. Second, endogenous stimulation with SERMs, aromatase inhibitors, or gonadotropins corrects endocrine parameters and is biologically rational in selected phenotypes, yet the best available evidence—dominated by observational cohorts at notable risk of bias—shows association rather than proven benefit for sperm retrieval, and large series find no effect of baseline testosterone or of the treatment response on retrieval, pregnancy, or live birth [69,89]. Third, and central to this review, the dynamic testosterone response is best understood as a prognostic biomarker of residual Leydig-cell functional reserve—and possibly of a more favourable testicular microenvironment—rather than a validated therapeutic target. On this basis, the author’s overall position is that the testosterone response should inform prognosis and patient selection rather than serve as a licence to treat, and that preoperative endocrine therapy belongs within clinical trials or carefully documented individualized care until randomized evidence resolves the biomarker-versus-target question.

## 12. Limitations and Future Directions

### 12.1. Limitations of the Evidence Base

The limitations of this field are substantial and must temper any recommendation. The evidence is dominated by retrospective designs with small samples and heterogeneous NOA etiologies. Definitions of hypogonadism, testosterone thresholds, assays, units (ng/dL versus nmol/L), drug protocols, and treatment durations vary widely, limiting comparability. Surgeon and laboratory expertise strongly influence retrieval and are rarely standardized, histology is inconsistently reported, and live-birth data are frequently absent. Selection bias and immortal-time bias, in which treated patients are by definition those who remained candidates over a treatment interval, can inflate apparent benefit. The overarching methodological problem, emphasized throughout this review, is the inability of observational data to determine whether the testosterone response identifies a biologically favorable testis or whether hormonal therapy itself improves retrieval [4,5,10].

### 12.2. Future Directions: From Empiricism to Precision Andrology

Resolving these questions requires prospective, ideally multicentre, randomized trials with standardized endocrine phenotyping, uniform testosterone and estradiol assays, and pre-specified delta-testosterone thresholds. Trials should stratify by histology and genetics and should be conducted separately for distinct phenotypes, including hypogonadal NOA, Klinefelter syndrome, hypergonadotropic NOA, low T/E2, and the salvage setting after failed micro-TESE. Composite endpoints spanning SRR, sperm quality, ICSI fertilization, embryo development, and live birth would prevent over-interpretation of endocrine surrogates, and validated nomograms incorporating dynamic endocrine variables would move the field from empiricism toward precision andrology [5,11]. Finally, for men in whom no spermatozoa can be retrieved, experimental strategies such as organotypic in vitro spermatogenesis and in vitro gametogenesis are under active investigation but remain pre-clinical and should be presented to patients as research rather than established options [102].

A further, non-hormonal direction is modulation of the retinoic-acid pathway, which is required for spermatogonial differentiation and meiotic initiation. Early clinical data suggest that isotretinoin may induce de novo ejaculated sperm in a subset of men with NOA or cryptozoospermia, particularly those with maturation arrest: in a single-center prospective series of 30 men, 37% developed reliable motile ejaculated sperm (54% among those with maturation-arrest histology), albeit with frequent dermatologic, metabolic, and neuropsychiatric adverse effects [103], and a 2026 systematic review and meta-analysis (6 studies, 225 men) reported de novo sperm in roughly 37–44% of azoospermic or cryptozoospermic men, at low-to-moderate certainty [104]. Because this evidence is preliminary and uncontrolled, retinoid-based therapy should currently be framed as investigational and hypothesis-generating rather than as part of routine pre-micro-TESE optimization. Mechanistically, this direction rests on the established role of retinoic acid as the key driver of spermatogonial differentiation and meiotic initiation in animal models [105], with the caveat that such preclinical findings do not translate directly to human retrieval outcomes.

Two further acquired or population-specific contexts merit brief mention. Gonadotoxic cancer therapy (notably alkylating agents) is an important acquired cause of NOA with its own endocrine trajectory and distinct fertility-preservation considerations, addressed in dedicated literature. The concept of impaired spermatogenesis also applies to transgender women, in whom gender-affirming oestrogen and antiandrogen therapy suppresses spermatogenesis and may render fertility-preservation counselling relevant; the SAGER guidelines provide the applicable standard for reporting sex and gender in such research [106]. A full account of either topic is beyond this review’s scope.

## 13. Conclusions

Exogenous testosterone should be avoided in fertility-seeking men because it suppresses the gonadotropin-dependent intratesticular environment required for spermatogenesis. In contrast, selected off-label strategies that stimulate endogenous testosterone production or support Sertoli-cell function are biologically rational before micro-TESE in carefully phenotyped men with NOA. Current evidence shows that hormonal optimization improves endocrine parameters and, in low-certainty analyses pooled largely from observational cohorts, is associated with—rather than proven to cause—higher sperm retrieval in selected men, particularly normogonadotropic and hypogonadal phenotypes; the data remain heterogeneous and insufficient to justify routine use. The dynamic rise in endogenous testosterone during treatment is an attractive functional biomarker of Leydig-cell reserve and possibly of a more favorable testicular microenvironment, yet it remains a candidate predictor rather than a validated decision-making tool. Reframed as phenotype-guided endocrine optimization combined with functional testing of Leydig-cell reserve, preoperative hormonal therapy in NOA can be positioned honestly: as an investigational, individualized adjunct that—consistent with current guideline advice to restrict its use before sperm retrieval to clinical trials—awaits definitive randomized evidence and should not yet be adopted as routine care.

## Figures and Tables

**Table 1:** Guideline statements on androgen and gonadotropin use in fertility-seeking men with NOA.

Organization	Statement	Strength	Implication for NOA
EAU	Do not use testosterone therapy for male infertility or in men wishing to father children [9]	Strong	Exogenous testosterone contraindicated; preserve spermatogenesis
AUA/ASRM	Do not prescribe exogenous testosterone to men interested in current or future fertility [7,8]	Clinical principle	Exogenous testosterone contraindicated
AUA/ASRM	May use aromatase inhibitors, hCG, or SERMs (alone or combined) in infertile men with low serum testosterone [7]	Conditional (Grade C)	Endogenous stimulation permissible in selected men
AUA/ASRM	Limited data support pharmacologic manipulation before surgical sperm retrieval in NOA [7,8]	Conditional (Grade C)	Counsel patients; treatment is off-label

**Table 2:** Pharmacologic options for preoperative hormonal optimization in NOA.

Therapy	Mechanism	Hormonal Effect	Best Candidate Phenotype	Evidence/Limitations
Clomiphene/SERM	Blocks estrogenic negative feedback; raises LH/FSH [4,27]	Higher endogenous testosterone	Low/normal gonadotropins; hypogonadal	Conflicting SRR data [5,43,44,45]
Anastrozole/letrozole (AI)	Inhibits testosterone-to-estradiol conversion [46]	Higher T and T/E2; lower E2	Low T/E2 ratio; obesity	Strong for semen params; weak for NOA SRR [46,47]
hCG	LH-receptor agonism on Leydig cells [27]	Higher intratesticular and serum T	Hypogonadism; selected primary NOA	Established in HH; observational in NOA [27,48]
FSH/hMG	Sertoli-cell stimulation [4,27]	Supports spermatogenesis	HH; selected late maturation arrest	Contested when endogenous FSH high [5,49]
Combinations	Engage Leydig and Sertoli signaling [4,27]	Composite	Selected, monitored cases	Higher cost; attribution difficult
Exogenous testosterone	Suppresses LH/FSH and intratesticular T [7,8,9]	Lower intratesticular T	CONTRAINDICATED in fertility seekers	Causes/worsens azoospermia

**Table 3:** Representative contemporary studies of hormonal therapy or testosterone dynamics before micro-TESE in NOA.

Study, Year [Ref]	Design	Population	Intervention	Key Result	Conclusion
Tharakan 2022 [5]	Systematic review/meta-analysis	NOA (22 studies, *n* = 1706)	Mixed hormonal therapy	OR 1.96 overall; normo OR 2.13 (*p* = 0.02), hyper OR 1.73 (*p* = 0.43)	Association (low-certainty), mainly normogonadotropic; not routine
Caroppo 2021 [4]	Narrative review + pooled	NOA	Various	Normal vs. low T: OR 1.63 (1.08–2.45)	Treat selected hypogonadal men
Peng 2022 [48]	Cohort	Hypergonadotropic NOA (*n* = 569)	hCG ± purified FSH	SRR 31.2% vs. 19.5%; aOR 1.59	Possible SRR benefit; no live-birth difference
Alrabeeah 2021 [43]	Retrospective	NOA (*n* = 122)	Clomiphene	SRR unaffected by clomiphene	No benefit
Vahidi 2023 [44]	Cross-sectional	NOA (*n* = 112)	Clomiphene 25 mg × 3 mo	25.9% vs. 31.0% (not significant)	No increase
Al-Zubi 2026 [45]	Retrospective	NOA (*n* = 152)	Clomiphene + hCG	SRR 45% vs. 50% (*p* = 0.53)	No significant benefit
Esteves 2024 [10]	Cohort	Hypogonadal NOA (*n* = 616)	Hormonal stimulation	SRR 56.6%; T 418.5 and delta-testosterone 258 ng/dL discriminate	Stimulation associated with SRR; causality not established
Barak 2026 [60]	Retrospective	NOA, low T (*n* = 77)	AI or rec-hCG	SRR 58%; AUC 0.785 for response	Hormonal response predicts SRR
Pelit 2025 [53]	Retrospective	Non-mosaic KS (*n* = 43)	Anastrozole vs. hCG-alfa	SRR 32.6%; anastrozole favored	Both raise T; AI better SRR
Raffo 2026 [61]	Multicentre cohort	Non-mosaic KS (*n* = 383)	TESE (real-life)	Positive retrieval 29.8%; higher total testosterone and hypospermatogenesis predictive	Baseline total testosterone and histology predict positive retrieval
Barak 2026 KS [62]	Retrospective/decision tree	Non-mosaic KS (*n* = 38)	rec-hCG	SRR 73.7%; T-change primary predictor	Decision model for counselling
Zachariou 2026 [63]	Cohort	HH-NOA (*n* = 35)	Gonadotropins	Ejaculated sperm 77%; micro-TESE 88%; 51% fatherhood	Etiologic therapy in HH
Ahmad 2025 [49]	Prospective	NOA (*n* = 20)	Recombinant gonadotropins	Benefit in late maturation arrest	Phenotype-dependent

**Table 4:** Studies evaluating testosterone dynamics as a predictor of sperm retrieval.

Study [Ref]	Population	Intervention	Response Metric	Association with SRR	Independent Predictor?
Reifsnyder 2012 [89]	NOA (*n* = 736 with hormonal data)	AI/clomiphene/hCG	Baseline T; response to therapy	None (56% vs. 52%, *p* = 0.29)	No
Esteves 2024 [10]	Hypogonadal NOA (*n* = 616)	Hormonal stimulation	Pre-op T 418.5; delta-testosterone 258 ng/dL	Positive (AUC 0.76–0.78)	Associated; causality not established
Barak 2026 [60]	NOA, low T (*n* = 77)	AI or rec-hCG	Pre-op T and T-change	Positive (AUC 0.785)	Yes (predictor)
Barak 2026 KS [62]	Non-mosaic KS (*n* = 38)	rec-hCG	T-change (decision tree)	Strong (up to ~97% vs. ~63%)	Yes (primary node)
Al-Zubi 2026 [45]	NOA (*n* = 152)	Clomiphene + hCG	Hormonal change	No SRR difference	No
Shiraishi 2021 [28]	NOA (*n* = 76; 4 given anastrozole)	Observational; anastrozole in subset	Intratesticular T/E2; Leydig-cell aromatase	Positive (lower ITT/higher ITE2 & higher aromatase; anastrozole raised ITT/ITE2)	Yes—aromatase independent correlate

## Data Availability

No new data were created or analyzed in this study. Data sharing is not applicable to this article.

## Abbreviations

The following abbreviations are used in this manuscript:

**AI**	aromatase inhibitor
**AMH**	anti-Müllerian hormone
**AR**	androgen receptor
**AUA/ASRM**	American Urological Association/American Society for Reproductive Medicine
**AUC**	area under the curve
**AZF**	azoospermia factor (regions AZFa, AZFb, AZFc)
**cAMP**	cyclic adenosine monophosphate
**CI**	confidence interval
**CYP11A1**	cholesterol side-chain-cleavage enzyme (P450scc)
**EAU**	European Association of Urology
**FSH**	follicle-stimulating hormone
**GAF**	Global Andrology Forum
**GnRH**	gonadotropin-releasing hormone
**hCG**	human chorionic gonadotropin
**HH**	hypogonadotropic hypogonadism
**hMG**	human menopausal gonadotropin
**HPG**	hypothalamic–pituitary–gonadal
**ICSI**	intracytoplasmic sperm injection
**ITT**	intratesticular testosterone
**KS**	Klinefelter syndrome
**LH**	luteinizing hormone
**micro-TESE**	microdissection testicular sperm extraction
**NOA**	non-obstructive azoospermia
**OR**	odds ratio
**PKA**	protein kinase A
**PRISMA**	Preferred Reporting Items for Systematic Reviews and Meta-Analyses
**SANRA**	Scale for the Assessment of Narrative Review Articles
**SERM**	selective estrogen receptor modulator
**SRR**	sperm retrieval rate
**StAR**	steroidogenic acute regulatory protein
**T**	testosterone
**T/E2**	testosterone-to-estradiol ratio
**TESE**	testicular sperm extraction
**WHO**	World Health Organization
